# Bis(2-amino-4-chloro­benzoato)triphenyl­anti­mony(V)

**DOI:** 10.1107/S160053680904358X

**Published:** 2009-10-28

**Authors:** Liyuan Wen, Handong Yin, Chuanhua Wang

**Affiliations:** a College of Chemistry and Chemical Engineering, Liaocheng University, Shandong 252059, People’s Republic of China

## Abstract

The title complex mol­ecule, [Sb(C_6_H_5_)_3_(C_7_H_5_ClNO_2_)_2_], possesses crystallographically imposed *C*_2_ symmetry. The Sb atom exhibits a trigonal-bipyramidal geometry with the axial positions occupied by the O atoms of two carboxyl­ate groups and the equatorial positions by the C atoms of the phenyl groups. Intra­molecular N—H⋯O and C—H⋯O hydrogen bonds occur.

## Related literature

For related structures, see: Yin *et al.* (2009 ▶); Ferguson *et al.* (1987 ▶); Rüther *et al.* (1985 ▶).
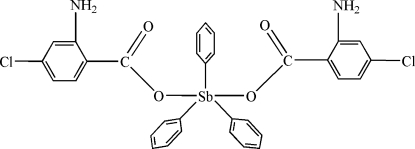


## Experimental

### 

#### Crystal data

[Sb(C_6_H_5_)_3_(C_7_H_5_ClNO_2_)_2_]
*M* *_r_* = 694.20
Orthorhombic, 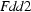

*a* = 13.0168 (13) Å
*b* = 20.298 (2) Å
*c* = 21.849 (3) Å
*V* = 5772.8 (11) Å^3^
*Z* = 8
Mo *K*α radiation
μ = 1.18 mm^−1^
*T* = 298 K
0.39 × 0.38 × 0.37 mm

#### Data collection

Siemens SMART CCD area-detector diffractometer
Absorption correction: multi-scan (*SADABS*; Sheldrick, 1996 ▶) *T*_min_ = 0.656, *T*_max_ = 0.669
5819 measured reflections
2493 independent reflections
2222 reflections with *I* > 2σ(*I*)
*R*_int_ = 0.019

#### Refinement

*R*[*F* ^2^ > 2σ(*F* ^2^)] = 0.021
*wR*(*F* ^2^) = 0.051
*S* = 1.11
2493 reflections
187 parameters
1 restraint
H-atom parameters constrained
Δρ_max_ = 0.35 e Å^−3^
Δρ_min_ = −0.25 e Å^−3^
Absolute structure: Flack (1983 ▶), 1181 Friedel pairs
Flack parameter: −0.02 (2)

### 

Data collection: *SMART* (Siemens, 1996 ▶); cell refinement: *SAINT* (Siemens, 1996 ▶); data reduction: *SAINT*; program(s) used to solve structure: *SHELXS97* (Sheldrick, 2008 ▶); program(s) used to refine structure: *SHELXL97* (Sheldrick, 2008 ▶); molecular graphics: *SHELXTL* (Sheldrick, 2008 ▶); software used to prepare material for publication: *SHELXTL*.

## Supplementary Material

Crystal structure: contains datablocks I, global. DOI: 10.1107/S160053680904358X/rz2373sup1.cif

Structure factors: contains datablocks I. DOI: 10.1107/S160053680904358X/rz2373Isup2.hkl

Additional supplementary materials:  crystallographic information; 3D view; checkCIF report

## Figures and Tables

**Table 1:** Hydrogen-bond geometry (Å, °)

*D*—H⋯*A*	*D*—H	H⋯*A*	*D*⋯*A*	*D*—H⋯*A*
N1—H1*A*⋯O2	0.86	2.07	2.704 (5)	130
C15—H15⋯O1	0.93	2.33	2.905 (4)	119

**Fig. 1.**
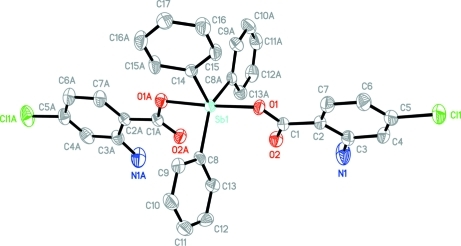
The molecular structure of the title compound, showing 50% probability displacement ellipsoids. H atoms are omitted for clarity. Symmetry code: (A) = -x, -y, z.

**Fig. 2.**
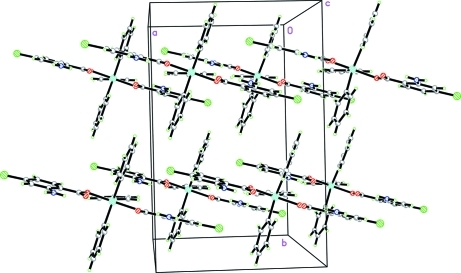
The crystal packing of the title compound.

## supplementary crystallographic information

### Comment

Organoantimony(V) complexes have been intensively studied owing to their versatile bonding modes (Yin *et al.*, 2009) and biological applications. We have therefore synthesized the title compound, and present its crystal structure here.

The molecular structure of the compound is shown in Fig.1. The complex molecule possesses crystallographically imposed *C*_2_ symmetry, the rotation axis passing through the Sb atom and bisecting the C14–C17/C15'/C16' phenyl ring. The coordination geometry around the five-coordinate antimony atom can be described as slightly distorted trigonal bipyramidal, with three C atoms of the phenyl groups occupying the equatorial positions and two O atoms of carboxylate groups at the axial positions. The average Sb—O bond length of 2.122 (2) Å is approximately equal to the sum of the covalent radii of Sb and O (2.07 Å), and lies within the range from 1.935 Å observed in triphenylstibine oxide (Ferguson *et al.*, 1987) to 2.506 Å found in tetraphenylstibonium benzenesulphonate hydrate (Rüther *et al.*, 1985). The Sb—C bond distances (Sb1—C8 = 2.101 (3) Å; Sb1—C8A = 2.101 (3) Å; Sb1—C14 = 2.122 (4) Å) fall in the normal range for Sb—*C*(phenyl) bonds (2.10–2.13 Å). The conformation of the complex molecule is enforced by intramolecular N—H···O and C—H···O hydrogen bonds (Table 1). The crystal packing (Fig. 2) is stabilized only by van der Waals interactions.

### Experimental

The reaction was carried out under nitrogen atmosphere. 2-amino-4-chlorobenzoic acid (1 mmol) and sodium ethoxide (1.2 mmol) were added to a stirred solution of methanol (30 ml) in a Schlenk flask and stirred for 0.5 h. Triphenylantimony dichloride (0.5 mmol) was then added to the reactor and the reaction mixture was stirred for 12 h at room temperature. The resulting clear solution was evaporated under vacuum. Colourless crystals suitable for X-ray analysis were obtained by slow evaporation of a ether/n-hexane (1:1 *v/v*) solution (yield 87%). Anal. Calcd (%) for C_32_H_25_Cl_2_N_2_O_4_Sb (Mr = 694.19): C, 55.37; H, 3.63; Cl, 10.21; N, 4.04. Found (%): C, 55.30; H, 3.74; Cl, 10.33; N, 4.16.

### Refinement

The C—H and N—H H atoms were positioned with idealized geometry and were refined isotropically using a riding model with N—H = 0.86 Å and C—H = 0.93 Å and with *U*_iso_(H) = 1.2 *U*_eq_(C, N).

### Crystal data

**Table:**

[Sb(C_6_H_5_)_3_(C_7_H_5_ClNO_2_)_2_]	*F*(000) = 2784
*M**_r_* = 694.20	*D*_x_ = 1.597 Mg m^−^^3^
Orthorhombic, *F**d**d*2	Mo *K*α radiation, λ = 0.71073 Å
Hall symbol: F 2 -2d	Cell parameters from 3849 reflections
*a* = 13.0168 (13) Å	θ = 2.7–26.8°
*b* = 20.298 (2) Å	µ = 1.18 mm^−^^1^
*c* = 21.849 (3) Å	*T* = 298 K
*V* = 5772.8 (11) Å^3^	Block, colourless
*Z* = 8	0.39 × 0.38 × 0.37 mm

### Data collection

**Table:**

Siemens SMART CCD area-detector diffractometer	2493 independent reflections
Radiation source: fine-focus sealed tube	2222 reflections with *I* > 2σ(*I*)
graphite	*R*_int_ = 0.019
φ and ω scans	θ_max_ = 25.0°, θ_min_ = 2.1°
Absorption correction: multi-scan (*SADABS*; Sheldrick, 1996)	*h* = −15→11
*T*_min_ = 0.656, *T*_max_ = 0.669	*k* = −24→22
5819 measured reflections	*l* = −25→24

### Refinement

**Table:**

Refinement on *F*^2^	Secondary atom site location: difference Fourier map
Least-squares matrix: full	Hydrogen site location: inferred from neighbouring sites
*R*[*F*^2^ > 2σ(*F*^2^)] = 0.021	H-atom parameters constrained
*wR*(*F*^2^) = 0.051	*w* = 1/[σ^2^(*F*_o_^2^) + (0.0236*P*)^2^ + 0.2508*P*] where *P* = (*F*_o_^2^ + 2*F*_c_^2^)/3
*S* = 1.11	(Δ/σ)_max_ < 0.001
2493 reflections	Δρ_max_ = 0.35 e Å^−^^3^
187 parameters	Δρ_min_ = −0.25 e Å^−^^3^
1 restraint	Absolute structure: Flack (1983), 1181 Friedel pairs
Primary atom site location: structure-invariant direct methods	Flack parameter: −0.02 (2)

### Fractional atomic coordinates and isotropic or equivalent isotropic displacement parameters (Å^2^)

**Table:**

	*x*	*y*	*z*	*U*_iso_*/*U*_eq_	
Sb1	0.0000	0.0000	0.024065 (19)	0.03974 (9)	
Cl1	0.64382 (7)	0.11557 (6)	0.05169 (7)	0.0943 (4)	
N1	0.3358 (3)	0.0520 (3)	0.17751 (19)	0.0964 (16)	
H1A	0.2733	0.0395	0.1828	0.116*	
H1B	0.3762	0.0559	0.2084	0.116*	
O1	0.15241 (14)	0.03663 (11)	0.01894 (11)	0.0483 (5)	
O2	0.15456 (18)	0.03090 (13)	0.12027 (11)	0.0561 (6)	
C1	0.1992 (2)	0.04151 (16)	0.07150 (17)	0.0450 (8)	
C2	0.3091 (2)	0.06030 (15)	0.06822 (16)	0.0448 (8)	
C3	0.3705 (3)	0.06552 (19)	0.12053 (19)	0.0576 (9)	
C4	0.4750 (3)	0.0831 (2)	0.1132 (2)	0.0675 (12)	
H4	0.5173	0.0862	0.1474	0.081*	
C5	0.5136 (3)	0.0953 (2)	0.0574 (3)	0.0626 (13)	
C6	0.4552 (4)	0.0923 (2)	0.0053 (2)	0.0665 (14)	
H6	0.4827	0.1026	−0.0329	0.080*	
C7	0.3533 (3)	0.0734 (2)	0.01176 (19)	0.0613 (10)	
H7	0.3130	0.0693	−0.0232	0.074*	
C8	−0.0531 (2)	0.09057 (16)	0.05826 (17)	0.0436 (8)	
C9	−0.0934 (2)	0.13384 (17)	0.01584 (19)	0.0552 (9)	
H9	−0.0985	0.1214	−0.0250	0.066*	
C10	−0.1264 (3)	0.19567 (18)	0.0339 (2)	0.0695 (11)	
H10	−0.1519	0.2251	0.0051	0.083*	
C11	−0.1215 (3)	0.2135 (2)	0.0937 (3)	0.0705 (12)	
H11	−0.1443	0.2549	0.1058	0.085*	
C12	−0.0830 (3)	0.1705 (2)	0.1366 (2)	0.0671 (13)	
H12	−0.0798	0.1832	0.1774	0.081*	
C13	−0.0492 (3)	0.10877 (18)	0.11947 (17)	0.0549 (9)	
H13	−0.0241	0.0796	0.1487	0.066*	
C14	0.0000	0.0000	−0.07304 (19)	0.0384 (10)	
C15	0.0908 (2)	0.0031 (2)	−0.10502 (17)	0.0586 (10)	
H15	0.1527	0.0057	−0.0839	0.070*	
C16	0.0907 (3)	0.0024 (2)	−0.16793 (18)	0.0699 (12)	
H16	0.1525	0.0036	−0.1893	0.084*	
C17	0.0000	0.0000	−0.1989 (2)	0.0627 (15)	
H17	0.0000	0.0000	−0.2415	0.075*	

### Atomic displacement parameters (Å^2^)

**Table:**

	*U*^11^	*U*^22^	*U*^33^	*U*^12^	*U*^13^	*U*^23^
Sb1	0.03262 (12)	0.04916 (15)	0.03746 (14)	0.00228 (16)	0.000	0.000
Cl1	0.0427 (5)	0.0907 (8)	0.1495 (13)	−0.0156 (5)	0.0057 (6)	−0.0279 (9)
N1	0.059 (2)	0.183 (5)	0.048 (3)	0.000 (3)	−0.0108 (18)	0.003 (3)
O1	0.0361 (10)	0.0658 (13)	0.0429 (13)	−0.0046 (10)	−0.0065 (11)	−0.0040 (13)
O2	0.0472 (13)	0.0755 (16)	0.0455 (14)	−0.0046 (12)	0.0037 (11)	0.0019 (13)
C1	0.0366 (16)	0.049 (2)	0.049 (2)	0.0028 (14)	−0.0051 (16)	−0.0044 (17)
C2	0.0375 (17)	0.0476 (19)	0.049 (2)	0.0028 (13)	−0.0048 (15)	−0.0028 (16)
C3	0.045 (2)	0.072 (2)	0.056 (2)	0.0052 (17)	−0.0088 (18)	−0.008 (2)
C4	0.048 (2)	0.072 (3)	0.082 (3)	0.0027 (18)	−0.021 (2)	−0.016 (2)
C5	0.044 (2)	0.057 (2)	0.087 (4)	−0.0050 (16)	0.005 (2)	−0.015 (2)
C6	0.048 (3)	0.085 (3)	0.067 (3)	−0.010 (2)	0.010 (2)	−0.008 (2)
C7	0.047 (2)	0.075 (3)	0.063 (3)	−0.0070 (18)	0.0008 (18)	−0.009 (2)
C8	0.0297 (15)	0.050 (2)	0.051 (2)	0.0031 (14)	0.0057 (15)	−0.0010 (17)
C9	0.0478 (18)	0.058 (2)	0.059 (2)	0.0043 (15)	−0.0086 (17)	−0.0025 (19)
C10	0.056 (2)	0.055 (2)	0.098 (4)	0.0121 (16)	−0.006 (2)	0.002 (3)
C11	0.052 (2)	0.057 (3)	0.103 (4)	0.0004 (19)	0.010 (2)	−0.013 (3)
C12	0.061 (2)	0.072 (3)	0.067 (3)	−0.005 (2)	0.012 (2)	−0.024 (3)
C13	0.053 (2)	0.059 (2)	0.053 (2)	0.0042 (16)	0.0027 (18)	−0.0049 (19)
C14	0.038 (2)	0.046 (2)	0.031 (2)	0.0020 (19)	0.000	0.000
C15	0.0319 (17)	0.099 (3)	0.045 (2)	−0.0012 (18)	−0.0007 (15)	−0.009 (2)
C16	0.046 (2)	0.119 (4)	0.045 (2)	0.004 (2)	0.0090 (17)	−0.003 (2)
C17	0.062 (3)	0.095 (4)	0.031 (3)	0.013 (3)	0.000	0.000

### Geometric parameters (Å, °)

**Table:**

Sb1—C8^i^	2.101 (3)	C7—H7	0.9300
Sb1—C8	2.101 (3)	C8—C9	1.381 (5)
Sb1—C14	2.122 (4)	C8—C13	1.388 (5)
Sb1—O1	2.1217 (19)	C9—C10	1.384 (5)
Sb1—O1^i^	2.1217 (19)	C9—H9	0.9300
Cl1—C5	1.749 (4)	C10—C11	1.357 (6)
N1—C3	1.352 (5)	C10—H10	0.9300
N1—H1A	0.8600	C11—C12	1.373 (6)
N1—H1B	0.8600	C11—H11	0.9300
O1—C1	1.304 (4)	C12—C13	1.380 (5)
O2—C1	1.233 (4)	C12—H12	0.9300
C1—C2	1.482 (4)	C13—H13	0.9300
C2—C7	1.387 (5)	C14—C15^i^	1.374 (4)
C2—C3	1.399 (5)	C14—C15	1.374 (4)
C3—C4	1.416 (5)	C15—C16	1.375 (5)
C4—C5	1.341 (7)	C15—H15	0.9300
C4—H4	0.9300	C16—C17	1.362 (5)
C5—C6	1.372 (6)	C16—H16	0.9300
C6—C7	1.389 (6)	C17—C16^i^	1.362 (5)
C6—H6	0.9300	C17—H17	0.9300
			
C8^i^—Sb1—C8	138.3 (2)	C2—C7—H7	118.6
C8^i^—Sb1—C14	110.83 (10)	C6—C7—H7	118.6
C8—Sb1—C14	110.83 (10)	C9—C8—C13	119.4 (3)
C8^i^—Sb1—O1	91.03 (10)	C9—C8—Sb1	116.3 (3)
C8—Sb1—O1	91.12 (10)	C13—C8—Sb1	124.3 (3)
C14—Sb1—O1	86.98 (6)	C8—C9—C10	120.2 (4)
C8^i^—Sb1—O1^i^	91.12 (10)	C8—C9—H9	119.9
C8—Sb1—O1^i^	91.03 (10)	C10—C9—H9	119.9
C14—Sb1—O1^i^	86.98 (6)	C11—C10—C9	120.1 (4)
O1—Sb1—O1^i^	173.95 (13)	C11—C10—H10	119.9
C3—N1—H1A	120.0	C9—C10—H10	119.9
C3—N1—H1B	120.0	C10—C11—C12	120.2 (4)
H1A—N1—H1B	120.0	C10—C11—H11	119.9
C1—O1—Sb1	114.6 (2)	C12—C11—H11	119.9
O2—C1—O1	121.9 (3)	C11—C12—C13	120.6 (4)
O2—C1—C2	122.8 (3)	C11—C12—H12	119.7
O1—C1—C2	115.3 (3)	C13—C12—H12	119.7
C7—C2—C3	118.4 (3)	C12—C13—C8	119.4 (4)
C7—C2—C1	119.5 (3)	C12—C13—H13	120.3
C3—C2—C1	122.1 (3)	C8—C13—H13	120.3
N1—C3—C2	123.1 (3)	C15^i^—C14—C15	118.9 (4)
N1—C3—C4	118.4 (4)	C15^i^—C14—Sb1	120.6 (2)
C2—C3—C4	118.4 (4)	C15—C14—Sb1	120.6 (2)
C5—C4—C3	120.6 (4)	C14—C15—C16	120.5 (3)
C5—C4—H4	119.7	C14—C15—H15	119.8
C3—C4—H4	119.7	C16—C15—H15	119.8
C4—C5—C6	122.7 (4)	C17—C16—C15	119.9 (4)
C4—C5—Cl1	118.1 (4)	C17—C16—H16	120.1
C6—C5—Cl1	119.2 (4)	C15—C16—H16	120.1
C5—C6—C7	117.2 (4)	C16—C17—C16^i^	120.4 (5)
C5—C6—H6	121.4	C16—C17—H17	119.8
C7—C6—H6	121.4	C16^i^—C17—H17	119.8
C2—C7—C6	122.7 (4)		

Symmetry codes: (i) −*x*, −*y*, *z*.

### Hydrogen-bond geometry (Å, °)

**Table:**

*D*—H···*A*	*D*—H	H···*A*	*D*···*A*	*D*—H···*A*
N1—H1A···O2	0.86	2.07	2.704 (5)	130
C15—H15···O1	0.93	2.33	2.905 (4)	119
